# A new alternative in plant retrograde signaling

**DOI:** 10.1186/gb4178

**Published:** 2014-05-30

**Authors:** Hou-Sung Jung, Todd C Mockler

**Affiliations:** 1 Biological Sciences, Dartmouth College, Hanover, NH 03755, USA; 2 Donald Danforth Plant Science Center, St Louis, MO 63132, USA

## Abstract

The reduced or oxidized state of plastoquinone in chloroplasts regulates splicing in the nucleus to control nuclear gene expression in response to changing environmental conditions.

## Introduction

In plants, chloroplasts and the nucleus communicate with each other to regulate growth and adapt to environmental conditions. Signaling from the nucleus to chloroplasts, by which the nucleus programs chloroplast development, is called anterograde regulation. In signaling from chloroplasts to the nucleus, or retrograde signaling, the chloroplasts send signals to the nucleus for regulation of nuclear gene expression [1]. Similar to mitochondria, chloroplasts are semi-autonomous organelles. During their evolution, the chloroplasts relinquished most of their genetic information to the nucleus. Modern chloroplast genomes encode only about 100 proteins. Meanwhile, it is predicted that more than 3,000 nuclear genes encode chloroplast proteins. Thus, chloroplasts need to coordinate gene expression in both chloroplasts and the nucleus to conduct their biological functions. In addition, besides the chloroplast proteins, other functional proteins such as stress response proteins are also under the control of retrograde signaling. This indicates that chloroplasts are sensors that respond to changing environmental conditions by regulating nuclear gene expression.

Nuclear gene expression is regulated in multiple steps including transcription, mRNA maturation, and translation. It has recently been reported that the mRNA maturation step is involved in plant responses to environmental conditions [2]. As RNA-Seq technologies advance, we can expect that more interesting findings, especially related to transcript maturation, will be discovered. In *Arabidopsis*, more than 60 % of intron-containing genes are alternatively spliced [3]. The frequency of alternative splicing (AS) further increases if different environmental conditions are included. AS transcripts can have both positive and negative effects on nuclear gene expression [4]. AS events provide diverse transcripts (and eventually proteins) from a single gene. This provides plants with the potential for greater plasticity, which is critical for plants to deal with fluctuating environmental conditions. This positive function of AS is supported by recent results [5]. However, AS is not always productive. AS events include exon skipping, alternative splice site selection and intron retention [3]. Among them, the most abundant AS type in plants is intron retention [3]. Transcripts containing intron retentions are futile in many cases because of the inclusion of a premature termination codon [4]. Thus, it has been speculated that AS may occur erroneously rather than following a programmed instruction [4]. More studies are required to determine the biological functions of most AS events in plants.

## Reduced and oxidized states of plastoquinone

Diverse chloroplast components have been proposed as the initiators of retrograde signaling. One of them is plastoquinone (PQ), a photosynthetic electron transport component [1]. PQ accepts electrons from the excited photosystem II (PSII) and becomes a reduced PQ (PQH_2_). PQH_2_ travels through the thylakoid membrane to find the cytochrome *b*_*6*_*f* complex (cyt *b*_*6*_*f*), a downstream component of PQ in the photosynthetic electron transport pathway. Following an electron donation to cyt *b*_*6*_*f*, PQH_2_ is oxidized to PQ.

The PQ’s reduced or oxidized (redox) state rapidly changes depending on environmental conditions. For example, when the light intensity is too low, the PQ pool is in an oxidized state because less PQ is reduced to PQH_2_. In contrast, a high light intensity causes an imbalance between generation and utilization of NADPH in chloroplasts. In this condition, PQH_2_ is more abundant than PQ and the PQ redox state is a reduced one.

## PQ redox state in retrograde signaling

About 20 years ago, Escoubas et al. [6] reported for the first time that the PQ redox state initiates retrograde signaling in order to change nuclear gene expression in an alga. It was later reported that the reduced PQ pool regulates stress-related nuclear gene expression in *Arabidopsis*[7]. More recent genomic studies have demonstrated that the PQ redox state has a global effect on nuclear gene expression [8,9]. Previous research [1] had shown the retrograde regulation of nuclear gene expression mainly at the level of transcriptional control. Petrillo *et al*. [10] recently demonstrated that the PQ redox state regulates AS of RNA-processing protein genes including the *ARGININE/SERINE-RICH SPLICING FACTOR 31* (*RS31*) gene, which encodes a plant Serine/Arginine-Rich (SR) domain splicing factor. The authors observed that a dark condition inhibited *RS31* mRNA maturation, leading to an increased level of *RS31* AS transcripts. The inhibition of *RS31* mRNA maturation was reversible because it was eliminated by light. After determining that *RS31* AS events were independent from light quality and photoreceptors, Petrillo *et al*. [10] turned their attention to modulation of chloroplast functional states in the dark. Using 3-(3,4-dichlorophenyl)-1,1-dimethylure (DCMU), which acts by blocking electron flow at the quinone acceptors of PSII, the authors showed that mRNA maturation was inhibited by the oxidized PQ state. Meanwhile, the reduced PQ state, which is induced by the chemical 2,5-dibromo-3-methyl-6-isopropylbenzoquinone (DBMIB), could reverse the splicing inhibition events. The oxidized PQ state could reproduce the effects of dark on *RS31* AS events, whereas the reduced PQ state resembled a light condition in terms of *RS31* AS status. All of these results indicate that the light/dark shift-dependent *RS31* AS is under the control of the PQ redox state. This is the first study reporting that the PQ redox state regulates the mRNA maturation steps for nuclear gene expression.

## Positive function of *RS31* alternative splicing

The *RS31* AS event reported by Petrillo *et al*. [10] is an example of a positive function of AS for gene expression regulation. In a light condition, *RS31* transcript is fully spliced and generates *mRNA1*, which encodes the functional RS31 protein. In a dark condition, however, AS generates *mRNA2* and *mRNA3*, at the expense of *mRNA1*. AS in the dark seems to be critical for this event because the authors determined that *RS31* transcript levels were not significantly changed during the light-to-dark shift. It is unlikely that *mRNA2* and *mRNA3* encode a functional full-length RS31 protein or any of its subdomains. This is because both *mRNA2* and *mRNA3* contain a premature translation termination codon and because it was shown that most of these transcripts are retained in the nucleus. To understand the biological function of the dark-induced AS of *RS31* from *mRNA1* to *mRNA2* and *mRNA3*, the authors generated a transgenic plant that constitutively expresses the *RS31 mRNA1*. The transgenic plant expresses a greater amount of the *RS31 mRNA1*, and is indistinguishable from wild-type plants in the light. In the dark, however, the over-accumulation of RS31 protein caused growth defect phenotypes. These results indicate that *Arabidopsis* uses AS to reduce the amount of RS31 protein levels to regulate plant development in light/dark shifts.

## Discussion

The report by Petrillo *et al*. [10] will give momentum to forthcoming experiments in the retrograde signaling field. First, what are the signaling molecules that can travel from shoot-to-root as mentioned in their report? The authors showed that most of the previously reported signaling candidate molecules, including reactive oxygen species, are unrelated to this PQ-dependent splicing event. Therefore, molecules that trigger this event remain to be discovered. Second, what intron-splicing components are regulated by the PQ redox state? Intron splicing from pre-mRNA requires many protein components. It would be interesting to investigate what components sense the PQ redox state in the nucleus. Third, a number of nuclear genes are under the control of the PQ redox state [8,9]. Do any of them undergo AS when they are induced by the PQ redox state? It is especially worth investigating the splicing variants of nuclear genes that are significantly induced by the PQ redox state. Finally, it would be interesting to know whether *RS31* AS will also be observed when the PQ redox state is induced by a shift between PSII- and PSI-specific light conditions [8]. In summary, Petrillo *et al*. [10] reported interesting phenomena that connect plants’ responses to changing light environments with the PQ redox state and AS events. Further studies will show how the PQ redox-driven retrograde signaling from chloroplasts regulates AS events in the nucleus. Ultimately, these findings can be applied to generate crop plants with enhanced adaptation to environmental conditions including abiotic stresses.

## Abbreviations

AS: Alternative splicing; NADPH: Reduced form of NADP^+^ (nicotinamide adenine dinucleotide phosphate); PQ: Plastoquinone; redox: reduced or oxidized.

## Competing interests

The authors declare that they have no competing interests.
